# Effects of rikkunshito on renal fibrosis and inflammation in angiotensin II-infused mice

**DOI:** 10.1038/s41598-019-42657-1

**Published:** 2019-04-17

**Authors:** Kengo Azushima, Kazushi Uneda, Hiromichi Wakui, Kohji Ohki, Kotaro Haruhara, Ryu Kobayashi, Sona Haku, Sho Kinguchi, Takahiro Yamaji, Shintaro Minegishi, Tomoaki Ishigami, Akio Yamashita, Kouichi Tamura

**Affiliations:** 1 Department of Medical Science and Cardiorenal Medicine, Yokohama City University Graduate School of Medicine, Yokohama, Japan; 2 Cardiovascular and Metabolic Disorders Program, Duke-NUS Medical School, Singapore, Singapore; 3 Department of Molecular Biology, Yokohama City University Graduate School of Medicine, Yokohama, Japan

**Keywords:** Kidney diseases, Pharmacology

## Abstract

The underlying pathogenesis of chronic kidney disease involves an activated renin-angiotensin system and systemic inflammation which ultimately develop renal injury. Rikkunshito (RKT) has been reported to exert anti-fibrotic and anti-inflammatory effects through enhancement of ghrelin signaling pathway. In this study, we investigated the effects of RKT on renal fibrosis and inflammation in angiotensin II (Ang II)-induced renal injury model. Ang II-infused mice exhibited hypertension, cardiac hypertrophy, increases in blood urea nitrogen and serum creatinine, moderate albuminuria and renal pathological changes such as mild urinary cast, interstitial macrophage infiltration and modest interstitial fibrosis. RKT had no evident effects on the Ang II-induced renal functional insufficiency and fibrosis, but attenuated renal interstitial macrophage infiltration. In addition, RKT significantly restored the Ang II-induced alteration in the expression of renal fibrosis- and inflammation-related genes such as type 3 collagen, transforming growth factor-β, monocyte chemoattractant protein-1 and interleukin-6. Furthermore, although RKT did not affect the expression of renal ghrelin receptor, an Ang II-induced decrease in renal sirtuin 1 expression, a critical down-stream pathway of the ghrelin receptor, was restored by RKT. These findings suggest that RKT potentially has a renal anti-inflammatory effect in the development of renal injury, and this effect could be mediated by the ghrelin signaling pathway.

## Introduction

Chronic kidney disease (CKD) is clinically characterized by the decline of glomerular filtration rate and presence of albuminuria^1^, in which the underlying pathogenesis involves an activated renin-angiotensin system (RAS) and systemic inflammation which ultimately develop renal injury^2^. Although aggressive risk factor management, such as blood pressure control and lifestyle/dietary modifications, has been recommended for CKD patients, the trend in prevalence of end stage renal disease is still increasing worldwide, resulting in a higher risk of cardiovascular diseases and mortality^3^. This clearly shows that new therapeutic strategies to treat CKD are unmet medical needs.

Rikkunshito (RKT), a traditional Japanese Kampo medicine, is an oral herbal drug that is often prescribed to treat patients with anorexia. Recently, it has been revealed that RKT has an agonistic effect on ghrelin signaling pathway by stimulating the secretion of ghrelin and enhancing the sensitivity of the ghrelin receptor, and exerts anti-fibrotic and anti-inflammatory effects^4,5^. Indeed, RKT has been reported to improve lung fibrosis and inflammation in bleomycin-treated mice and prolong survival in a mouse model of aging through enhancement of the ghrelin signaling pathway^6,7^. Ghrelin, an orexigenic hormone mainly secreted from the stomach in response to caloric restriction, has recently been highlighted to have anti-inflammatory effects and become considered as a new therapeutic candidate for cardiovascular and renal diseases^8–10^. Several previous studies have reported that ghrelin replenishment therapy improves renal function and fibrosis along with the suppression of inflammation in rodent models of kidney diseases and injuries^11–15^. These findings suggest that, as one of agonistic agents in the ghrelin axis, RKT also could exert beneficial effects against the development of kidney diseases and injuries. Nevertheless, the effects of RKT on kidney diseases and injuries have never been investigated.

In this study, we investigated whether RKT could exert anti-fibrotic and anti-inflammatory effects in the development of renal injury using angiotensin II (Ang II)-induced renal injury model. We examined the effects of RKT on renal function and pathological changes, fibrosis- and inflammation-related gene expression and the ghrelin signaling pathway.

## Results

### Physiological parameters in vehicle, Ang II and Ang II + RKT groups

**Figure 1:**
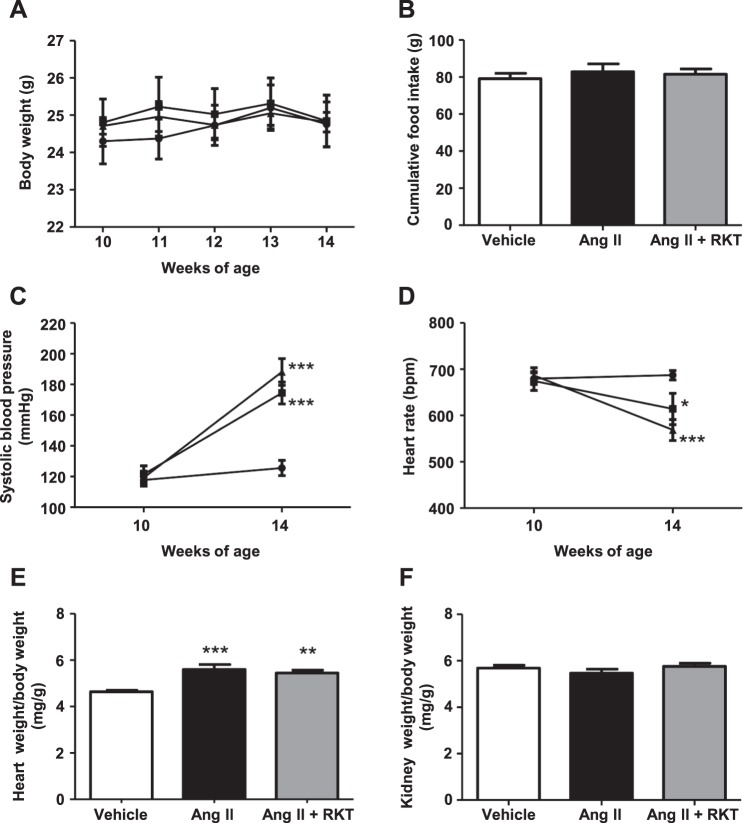
Effects of vehicle, Ang II and Ang II + RKT treatments on body weight, food intake, systolic blood pressure, heart rate and tissue weight. (**A**) Body weight change in vehicle, Ang II and Ang II + RKT groups (n = 7–8). ●, vehicle group; ■, Ang II group; ▲, Ang II + RKT group. (**B**) Cumulative food intake in vehicle, Ang II and Ang II + RKT groups (n = 7). (**C,D**) Time courses of systolic blood pressure and heart rate in vehicle, Ang II and Ang II + RKT groups (n = 7–8). ●, vehicle group; ■, Ang II group; ▲, Ang II + RKT group. (**E,F**) Heart weight/body weight and kidney weight/body weight ratios in vehicle, Ang II and Ang II + RKT groups (n = 6–8). (**A,C,D**) *P < 0.05, ***P < 0.001, vs vehicle group. Data were analyzed by two-way repeated measures ANOVA. (**B,E and F**) **P < 0.01, ***P < 0.001, vs vehicle group. Data were analyzed by one-way ANOVA. Ang II, angiotensin II; RKT, rikkunshito.

At 10 weeks of age when the experiment started, body weight, systolic blood pressure and heart rate were identical between the three groups. The body weight change and cumulative food intake during the experimental period were comparable between the three groups (Fig. 1A,B). At 14 weeks of age, systolic blood pressures in the Ang II and Ang II + RKT groups were significantly and similarly increased compared with that of the vehicle group (Fig. 1C). In contrast, heart rates in the Ang II and Ang II + RKT groups were significantly and similarly decreased compared with that of the vehicle group (Fig. 1D). The heart weight and body weight ratio was significantly and similarly increased in the Ang II and Ang II + RKT groups compared with that of the vehicle group, whereas the kidney weight and body weight ratio was identical between the three groups (Fig. 1E,F).

### RKT treatment has no evident effects on Ang II-induced renal functional insufficiency

**Figure 2:**
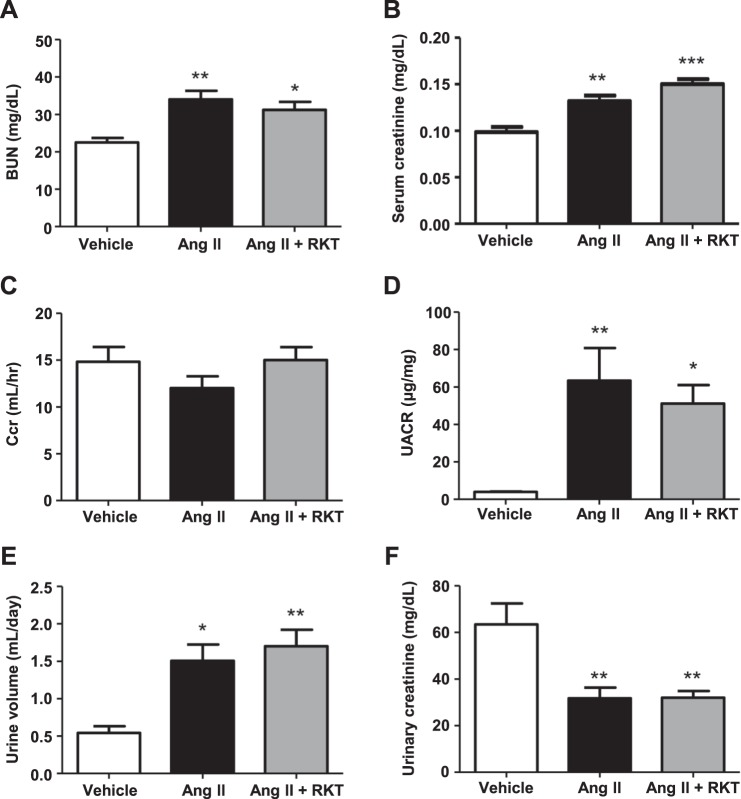
Effects of vehicle, Ang II and Ang II + RKT treatments on renal function. (**A**) BUN in vehicle, Ang II and Ang II + RKT groups (n = 6–7). (**B**) Serum creatinine in vehicle, Ang II and Ang II + RKT groups (n = 6–7). **(C)** Ccr in vehicle, Ang II and Ang II + RKT groups (n = 6–7). **(D)** UACR in vehicle, Ang II and Ang II + RKT groups (n = 6–7). **(E)** Urine volume in vehicle, Ang II and Ang II + RKT groups (n = 6–7). **(F)** Urinary creatinine in vehicle, Ang II and Ang II + RKT groups (n = 6–7). *P < 0.05, **P < 0.01, ***P < 0.001, vs vehicle group. Data were analyzed by one-way ANOVA. Ang II, angiotensin II; RKT, rikkunshito; BUN, blood urea nitrogen; Ccr, creatinine clearance; UACR, urinary albumin-to-creatinine ratio.

To estimate the effects of RKT on the Ang II- induced kidney injury, we firstly examined renal functions. Blood urea nitrogen (BUN) and serum creatinine in the Ang II and Ang II + RKT groups were significantly and similarly increased compared with those of the vehicle group (Fig. 2A,B). Although RKT tended to slightly attenuate the Ang II-induced decrease in creatinine clearance (Ccr) and increase in urinary albumin-to-creatinine ratio (UACR), these changes were not statistically significant (Fig. 2C,D). Urine volume in the Ang II and Ang II + RKT groups were significantly and similarly increased compared with those of the vehicle group, whereas urinary creatinine in the Ang II and Ang II + RKT groups were significantly and similarly decreased (Fig. 2E,F).

### RKT treatment attenuates Ang II-induced renal interstitial macrophage infiltration

**Figure 3:**
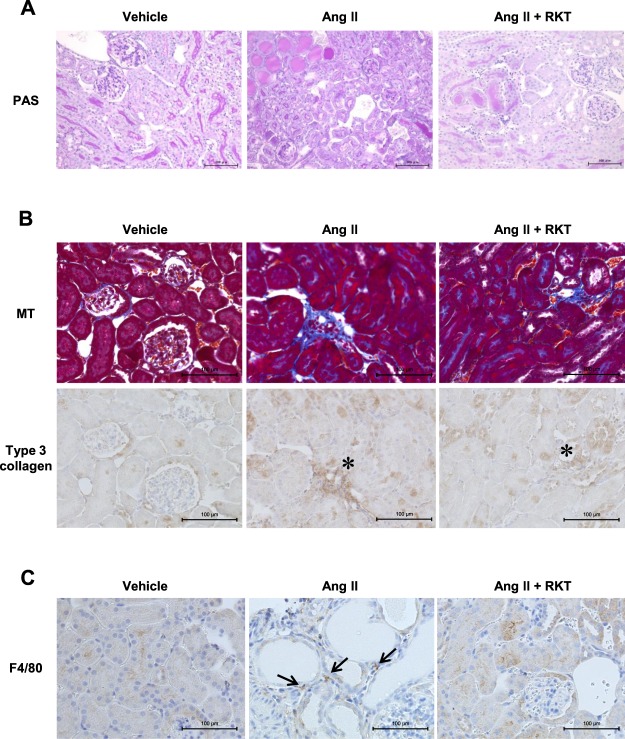
Effects of vehicle, Ang II and Ang II + RKT treatments on renal histopathological changes. (**A**) Representative images of PAS-stained kidney sections in vehicle, Ang II and Ang II + RKT groups (original magnification, ×200; bar, 100 μm). **(B)** Representative images of MT- and an anti-type 3 collagen antibody-stained consecutive kidney sections in vehicle, Ang II and Ang II + RKT groups (original magnification, ×400; bar, 100 μm; asterisk, enhanced region of the anti-type 3 collagen antibody). **(C)** Representative images of an anti-F4/80 antibody-stained kidney sections in vehicle, Ang II and Ang II + RKT groups (original magnification, ×400; bar, 100 μm; arrow, F4/80 positive cell). Ang II, angiotensin II; RKT, rikkunshito; PAS, periodic acid-schiff; MT, masson trichrome.

Next, we examined renal histopathological changes. Compared with the vehicle group, mild urinary cast was observed in both the Ang II and Ang II + RKT groups (Fig. 3A). In addition, consecutive sections stained with Masson’s trichrome and an antibody against type 3 collagen showed that, compared with the vehicle group, modest renal interstitial fibrosis was observed in both the Ang II and Ang II + RKT groups (Fig. 3B). However, there was no obvious difference in these renal structural changes such as urinary cast and renal interstitial fibrosis between the Ang II and Ang II + RKT groups (Fig. 3A,B). To investigate the effect of RKT on renal macrophage infiltration, we further performed immunostaining using an antibody against F4/80. Although mild infiltration of F4/80 positive cells in renal interstitial tissue was observed in the Ang II group, this infiltration was not observed in the vehicle and Ang II + RKT groups (Fig. 3C).

### RKT treatment attenuates Ang II-induced increase in renal fibrosis- and inflammation-related gene expression

**Figure 4:**
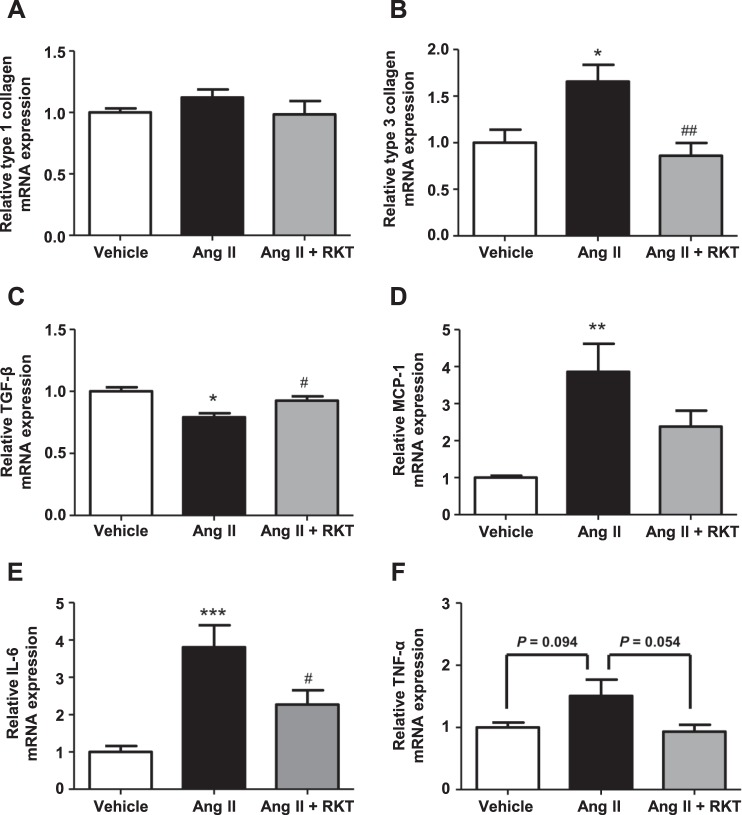
Effects of vehicle, Ang II and Ang II + RKT treatments on the renal mRNA expression of fibrosis and inflammatory markers. (**A–C**) Renal mRNA expression of fibrosis markers (type 1 collagen, type 3 collagen and TGF-β) in vehicle, Ang II and Ang II + RKT groups (n = 5–8). (**D–F**) Renal mRNA expression of inflammatory makers (MCP-1, IL-6 and TNF-α) in vehicle, Ang II and Ang II + RKT groups (n = 6–8). *P < 0.05, **P < 0.01, ***P < 0.001, vs vehicle group. ^#^*P* < 0.05, ^##^*P* < 0.01, vs Ang II group. Data were analyzed by one-way ANOVA. Ang II, angiotensin II; RKT, rikkunshito; TGF-β, transforming growth factor-β; MCP-1, monocyte chemoattractant protein-1; IL-6, interleukin-6; TNF-α, tumor necrosis factor-α.

To further investigate the effects of RKT in the kidney of Ang II-infused mice, we examined renal fibrosis- and inflammation-related gene expression. Regarding fibrosis-related genes, although the renal expression of type 1 collagen was comparable between the three groups (Fig. 4A), the renal expression of type 3 collagen was significantly increased in the Ang II group compared with the vehicle group, and this increase was significantly attenuated in the Ang II + RKT group (Fig. 4B). In contrast, the renal expression of transforming growth factor-β (TGF-β) was significantly decreased in the Ang II group compared with the vehicle group, and this decrease was significantly attenuated in the Ang II + RKT group (Fig. 4C). Regarding inflammation-related genes, the renal expressions of monocyte chemoattractant protein-1 (MCP-1) and interleukin-6 (IL-6) were significantly increased in the Ang II group compared with the vehicle group, and these increases were attenuated in the Ang II + RKT group (Fig. 4D,E). In addition, although it was not statistically significant, the renal expression of tumor necrosis factor-α (TNF-α) showed a trend toward increase in the Ang II group compared with the vehicle group, and this increase showed a trend toward attenuation in the Ang II + RKT group (Fig. 4F).

### Ghrelin signaling pathway could be modulated by RKT treatment

**Figure 5:**
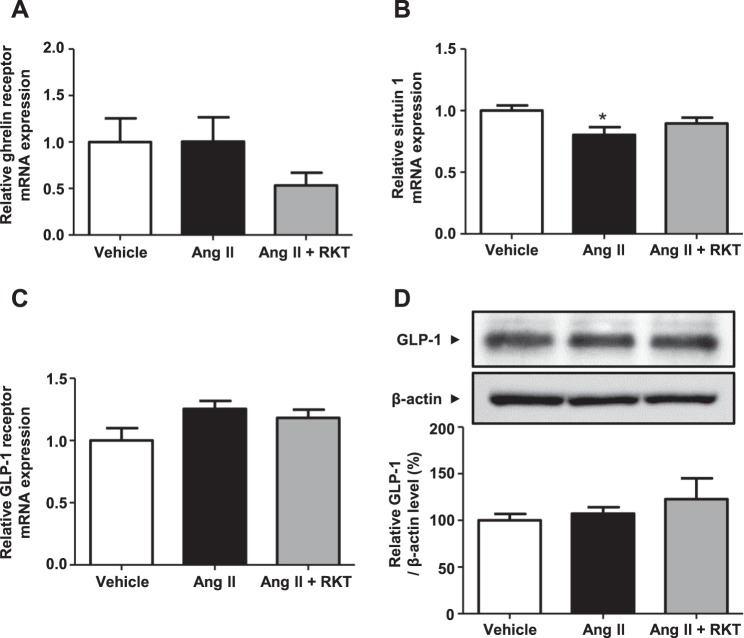
Effects of vehicle, Ang II and Ang II + RKT treatments on renal ghrelin signaling pathway. (**A**) Renal mRNA expression of ghrelin receptor in vehicle, Ang II and Ang II + RKT groups (n = 6–7). **(B)** Renal mRNA expression of sirtuin 1 in vehicle, Ang II and Ang II + RKT groups (n = 6–7). **(C)** Renal mRNA expression of GLP-1 receptor in vehicle, Ang II and Ang II + RKT groups (n = 6–7). **(D)** Representative western blots and quantitative analysis of renal GLP-1 protein levels in vehicle, Ang II and Ang II + RKT groups (n = 6–7). *P < 0.05, vs vehicle group. Data were analyzed by one-way ANOVA. Ang II, angiotensin II; RKT, rikkunshito; GLP-1, glucagon-like peptide-1.

To investigate the possible mechanism involved in the improvement of renal fibrosis- and inflammation-related gene expression in the Ang II + RKT group, we examined the renal ghrelin signaling pathway. Although expression of the ghrelin receptor was not significantly different between the three groups (Fig. 5A), the expression of sirtuin 1, which has recently been reported as a critical down-stream signaling pathway of the ghrelin receptor^4,5^, was significantly decreased in the Ang II group compared with the vehicle group. Ang II-induced down-regulation of renal sirtuin 1 expression was restored by RKT treatment (Fig. 5B). As an alternative pathway that could affect sirtuin 1 expression, we also investigated glucagon-like peptide-1 (GLP-1) axis^16,17^. As shown in Fig. 5C,D, the renal expression of GLP-1 receptor and the renal protein level of GLP-1 were comparable between the three groups.

## Discussion

This is the first study to investigate the beneficial effects of RKT against the development of renal injury. In this study, we showed that RKT had no evident effects on Ang II-induced renal functional insufficiency and fibrosis, but attenuated renal interstitial macrophage infiltration. In addition, RKT significantly restored the Ang II-induced alteration in the expression of renal fibrosis- and inflammation-related genes. Furthermore, the Ang II-induced decrease in renal sirtuin 1 expression, a critical down-stream signaling pathway of the ghrelin receptor, was attenuated in the Ang II + RKT group. These findings suggest that RKT potentially exerts renal anti-inflammatory effects in the development of renal injury, and these effects may be mediated by the renal ghrelin signaling pathway.

In this study, Ang II-infused mice exhibited hypertension, cardiac hypertrophy, increases in BUN and serum creatinine, moderate albuminuria and renal pathological changes such as mild urinary cast, interstitial macrophage infiltration and modest interstitial fibrosis, along with an increase in renal fibrosis- and inflammation-related gene expression. These findings are consistent with previous studies demonstrating that the Ang II-infused mouse was an activated RAS model that exhibits organ damage, such as hypertension, cardiac/vascular hypertrophy and renal functional insufficiency, concomitant with increases in pro-fibrotic/inflammatory genes and oxidative stress^18^.

Ghrelin, an orexigenic hormone secreted mainly from the stomach, plays an important role in the regulation of food intake and adiposity^19^. Ghrelin replenishment is reported to elicit a significant increase in food intake by exerting a potent appetite-stimulating effect through altering orexigenic neuropeptides in hypothalamus^20,21^. Regarding the potential mechanism and ingredients of RKT which could activate the ghrelin signaling pathway, atractylodin, hesperidin/heptamethoxyflavone and 10-gingerol, which derive from *Atractylodis lanceae rhizome*, *Aurantii nobilis pericarpium* and *Zingiberis rhizoma* respectively, has been reported as active components^4^. Several previous studies have reported that atractylodin enhances ghrelin receptor signaling by sensitizing the ghrelin receptor, hesperidin/heptamethoxyflavone promotes ghrelin secretion via 5-HT2b/c receptors, and 10-gingerol inhibits the ghrelin deacylation^22–24^. Therefore, RKT is considered to exert its agonistic effect on the ghrelin signaling pathway through a synergitic action of various ingredients. However, the body weight change and cumulative food intake in this study were comparable between the three experimental groups. RKT has been reported to exert its orexigenic effect in mice with anorexia caused by exposure to novelty stress, chemotherapy and surgery^6,25–28^. In contrast, in mice without interventions that cause anorexia, RKT failed to exert the orexigenic effect such as increases of food intake and body weight change, even though the ghrelin signaling pathway was enhanced^7,29^. These findings suggest that RKT tends to exert its orexigenic effect under the condition of anorexia. In this study, since the Ang II-infused mice did not exhibit anorexia, RKT may not affect their food intake and body weight change.

The RKT treatment suppressed an Ang II-induced increase in renal type 3 collagen, MCP-1, IL-6 and TNF-α expression. These results are consistent with a previous study demonstrating that ghrelin replenishment therapy suppresses Ang II-induced renal injuries along with the reduction of renal oxidative stress and inflammation^12^. In contrast, in this study, renal TGF-β expression was significantly decreased by Ang II- infusion, and this decrease was significantly restored by RKT. Although TGF-β is generally considered as a central regulator of renal fibrosis, it also acts as an anti-inflammatory factor in kidney diseases^30–32^. Previous studies have shown that targeted deletion of the TGF-β1 gene in mice results in the development of a lethal inflammatory response in many tissues^33^, whereas mice overexpressing latent TGF-β1 are protected against the development of renal inflammation in several kidney disease models^34,35^. Therefore, the result of renal TGF-β expression in this study may reflect the degree of renal inflammation in the respective experimental groups. In the renal histological analyses, RKT attenuated the Ang II-induced renal interstitial macrophage infiltration, which is consistent with the result that RKT suppressed the Ang II-induced increase in the expression of inflammation-related genes. These findings indicate that RKT suppresses the Ang II-induced renal tissue inflammation, which in turn contributes to the attenuation of macrophage infiltration. On the other hand, RKT had no evident effects on the Ang II-induced renal interstitial fibrosis despite the significant suppression of type 3 collagen expression in the Ang II + RKT group. This discrepancy might be caused by the degree of renal fibrosis induced by Ang II-infusion. Since the 4-week Ang II-infusion elicited the very early stage of renal fibrosis, it might be difficult to histologically estimate the effects of RKT on renal fibrosis. Studies using longer Ang II- infusion and unilateral ureteral obstruction would be necessary to address this issue.

Notably, the favorable effect of RKT on renal inflammation was accompanied by the attenuation of an Ang II-induced decrease in renal sirtuin 1 expression. Recently, it has been revealed that sirtuin 1 is a critical down-stream pathway of the ghrelin receptor^4,5^; several *in vitro* studies have reported that ghrelin treatment elevates sirtuin 1 expression or activity in human umbilical vein endothelial cells, retinal microvascular endothelial cells and cortical collecting duct cells^7,36,37^. In addition, Fujitsuka *et al*. have reported that RKT activates sirtuin 1 via the ghrelin receptor, which in turn contributes to protective effects against brain and other organ pathologies, such as inflammation and apoptosis, in models of accelerated senescence, klotho-deficient and senescence-accelerated mouse prone/8 mice^7^. These findings suggest that enhancement of the ghrelin receptor/sirtuin 1 pathway is one of possible mechanisms of the favorable effect of RKT on renal inflammation in the Ang II-induced renal injury model.

The following limitations are necessary to be considered in this study. First, a vehicle + RKT group is lack in this study. Therefore, it is unknown whether RKT has any effects on the kidney in normal mice. Second, because RKT treatment did not improve the Ang II-induced renal functional insufficiency, it is still unknown whether RKT has actual reno-protective effects against the development of renal injury. This absence of effect may have resulted from the experimental design such as the experimental period, doses of Ang II and RKT, or the renal injury model. Studies using longer Ang II- infusion and other renal injury models, such as adenine- infusion, 5/6 nephrectomy, would be necessary to estimate the actual reno-protective effect of RKT. Third, we have not examined direct evidence that RKT enhances the ghrelin signaling pathway. Further studies using ghrelin receptor inhibitors and ghrelin knock-out mice are necessary to address this issue. Fourth, the Ang II-infusion induced significant increases in BUN and serum creatinine in the Ang II and Ang II + RKT groups which is compatible with renal functional insufficiency, but there is no significant difference in Ccr between the three groups. This discrepancy may be caused by a renal creatinine excretion in mice. The renal creatinine excretion in mice is markedly higher than that in human, which contributes to the poor validity of Ccr as a method estimating the glomerular filtration rate (GFR) in mice^38,39^. Further study using inulin clearance would be necessary to accurately examine GFR in this model.

In conclusion, RKT had no evident effects on renal functional insufficiency and fibrosis in Ang II-infused mice. Nonetheless, RKT attenuated the Ang II-induced renal interstitial macrophage infiltration along with the significant suppression of the Ang II-induced increase in renal inflammation-related genes. Furthermore, the Ang II-induced decrease in renal sirtuin 1 expression, a critical down-stream pathway of the ghrelin receptor, was restored by RKT. These results suggest that, although the anti-fibrotic effect of RKT still remains to be elucidated, RKT potentially exerts renal anti-inflammatory effects in the development of renal injury, and these effects may be mediated by the renal ghrelin signaling pathway. Further studies are necessary to elucidate the actual reno-protective effects of RKT and the details of its mechanism to determine if RKT has potential as a new therapeutic candidate to treat CKD patients.

## Methods

### Animals and treatments

This study was performed in accordance with the National Institutes of Health guidelines for the use of experimental animals. All animal studies were reviewed and approved by the Animal Studies Committee of Yokohama City University, Japan. All animals were housed in an air-conditioned room (25 °C) with a 12-hour light-dark cycle and were allowed free access to food and water.

To make a kidney injury in mice, we employed 129/Sv strain. The 129/Sv strain has been reported to have a susceptibility to the development of hypertension and kidney injuries caused by 5/6 nephrectomy compared with C57BL/6 strain, and this susceptibility is considered to be associated with the activated RAS in the 129/Sv strain^40,41^. Male 129/SvJcl mice at the age of 10 weeks were divided into three groups as follows. 1) saline-infused mice fed a standard diet (0.6% NaCl and 3.4 kcal/g, CE-2; CLEA, Japan), vehicle group; 2) Ang II (500 ng/kg/min)-infused mice fed a standard diet, Ang II group; 3) Ang II (500 ng/kg/min)-infused mice fed a diet containing RKT (CE-2 containing 1.5% RKT), Ang II + RKT group. Osmotic mini-pumps (Model 2004; ALZET, USA) were subcutaneously implanted in mice in all groups^42,43^, and saline and Ang II were administered for 4 weeks. The dose of Ang II was determined in accordance with the results of our preliminary experiments (500 and 1000 ng/kg/min). During the experimental period, body weight and food intake were measured weekly, as described previously^44^. At the end of the experimental period (14 weeks of age), mice were sacrificed in the fed state between 10:00 and 14:00. Collected blood samples and tissues were immediately frozen by liquid nitrogen and stocked at -80 °C until use.

The Kampo medicine RKT is a dried powder from the hot water extract composed of eight herbal drugs; Atractylodes lancea rhizome (*Atractylodis lanceae rhizoma*), Ginseng (*Ginseng radix*), Pinellia Tuber (*Pinellia tuber*), Poria Sclerotium (*Poria*), Jujube (*Zizyphi fructus*), Citrus Unshiu Peel (*Aurantii nobilis pericarpium*), Glycyrrhiza (*Glycyrrhizae radix*) and Ginger (*Zingiberis rhizoma*). The study diet was prepared by mixing the powdered diet (CE-2; CLEA Japan) with RKT at a concentration of 1.5%. This concentration of RKT is equivalent to the amount of 2000 mg/kg/day that was determined by referring to the protocol of previous studies^7,28,29^. RKT was obtained from Tsumura & Co. (Japan).

### Metabolic cage analysis

Metabolic cage analysis was performed as previously described^45,46^. Mice were given free access to water and fed the standard diet and the diet containing RKT. A 24-hour urine collection was performed at the end of the experimental period.

### Blood pressure and heart rate measurements

Systolic blood pressure and heart rate were measured by the tail-cuff method (BP- monitor MK-2000; Muromachi Kikai Co., Japan), as described previously^47,48^. This system measures blood pressure in conscious mice without any preheating. All measurements were performed between 10:00–14:00. At least eight measurements were performed in each mouse and the average of the measurements was used for the analysis. Blood pressure and heart rate were measured at the start and end of the experimental period.

### Biochemical assays

Blood samples were collected by cardiac puncture when mice were sacrificed, as described previously^46,49^. Whole blood was centrifuged at 800 × g, 4 °C for 15 min to separate the plasma. The resulting plasma was stored at −80 °C until use. Plasma creatinine and urinary creatinine were measured using an autoanalyzer (Hitachi 7180; Hitachi, Japan). Urinary albumin was measured by an immunoturbidimetric assay (FUJIFILM WAKO Pure Chemical Corporation, Japan).

### Histological analysis

Histological analysis was performed as described previously^43,50^. The kidneys were fixed with 4% paraformaldehyde overnight and embedded in paraffin. Sections (4 μm thick) were stained with periodic acid–Schiff and Masson’s trichrome. Paraffin sections of kidney tissue were also stained with antibodies against F4/80 (rat monoclonal; Abcam, Japan) and type 3 collagen (rabbit polyclonal; Abcam, Japan), as described previously^49^. Briefly, after antigen retrieval was performed by microwave heating, the sections were blocked for endogenous biotin activity using peroxidase blocking reagent (DAKO) and treated for 60 minutes with 10% normal goat serum in phosphate-buffered saline. The sections were then incubated with the anti-F4/80 antibody (diluted 1:100) and anti-type 3 collagen antibody (diluted 1:100) at room temperature for 2 hours. Morphometric analysis was performed using a BZ-9000 fluorescence microscope (Keyence, Japan).

### Real-time quantitative RT-PCR analysis

Total RNAs were extracted from kidney with ISOGEN (Nippon Gene Co., LTD., Japan), and the cDNA was synthesized using the SuperScript III First-Strand System (Invitrogen, USA). Real-time quantitative RT-PCR was performed with an ABI PRISM 7000 Sequence Detection System by incubating the reverse transcription product with TaqMan PCR Master Mix and a designed Taqman probe [collagen-1α, Mm00801666_g1; collagen-3α, Mm01254476_m1; TGF-β, Mm01178820_m1; MCP-1, Mm_00441242_m1; IL-6, Mm_00446190_m1; TNF-α, Mm00443258_m1; ghrelin receptor, Mm00616415_m1; sirtuin1, Mm00490758_m1; Glp1r, Mm00445292_m1; Applied Biosystems, USA], essentially as described previously^46^. The mRNA levels were normalized to those of the 18 S rRNA control.

### Immunoblot analysis

Immunoblot analysis was performed as described previously^48,51^. Briefly, total protein extract was performed from kidney tissues with sodium dodecyl sulfate-containing sample buffer. The protein concentration of each sample was measured using a Detergent Compatible Protein Assay Kit (Bio-Rad, Japan). Equal amounts of protein extract were fractionated on a 5–20% polyacrylamide gel (ATTO, Japan). The gel was then transferred to a polyvinylidene difluoride (PVDF) membrane using an iBlot Dry Blotting System (Invitrogen, USA). Membranes were blocked for 1 hour at room temperature with phosphate-buffered saline containing 5% skim milk powder, and were then probed overnight at 4 °C with a specific primary antibody to GLP-1(Abcam, Japan). Membranes were washed and further incubated with secondary antibodies for 15 minutes at room temperature. The sites of the antibody–antigen reaction were visualized by enhanced chemiluminescence substrate (GE Healthcare, Japan). Images were analyzed quantitatively using a Fuji LAS-3000 image analyzer (Fuji Film, Japan).

### Statistical analysis

Statistical analyses were performed using GraphPad Prism software (GraphPad Software, La Jolla, CA, USA). All data are shown as the mean ± SEM. Differences were analyzed as follows. A one-way ANOVA with Tukey post-test was used to test for differences between treatment groups. A two-way repeated measures ANOVA was used to test for differences over time. Values of *P* < 0.05 were considered statistically significant.

## Supplementary information

Dataset 1

## Data Availability

All relevant data are within the paper. The datasets are available from the corresponding authors with reasonable request.
