# Supplementary material for: Effects of rikkunshito on renal fibrosis and inflammation in angiotensin II-infused mice
Source: Sci Rep. 2019 Apr 17;9:6201. doi: 10.1038/s41598-019-42657-1 (PMC6470237; doi:10.1038/s41598-019-42657-1)
Supplement: Supplementary file 1 — Dataset 1 [file 41598_2019_42657_MOESM1_ESM.pdf]

**Effects of rikkunshito on renal fibrosis and inflammation in angiotensin II-infused mice**

Kengo Azushima[1,2†*], Kazushi Uneda[1†], Hiromichi Wakui[1*], Kohji Ohki[1], Kotaro Haruhara[1], Ryu Kobayashi[1], Sona Haku[1], Sho Kinguchi[1], Takahiro Yamaji[1], Shintaro Minegishi[1], Tomoaki Ishigami[1], Akio Yamashita[3] and Kouichi Tamura[1]

[†] These authors contributed equally to this work.

[1] Department of Medical Science and Cardiorenal Medicine, Yokohama City University Graduate School of Medicine, Yokohama, Japan

[2] Cardiovascular and Metabolic Disorders Program, Duke-NUS Medical School, Singapore

[3] Department of Molecular Biology, Yokohama City University Graduate School of Medicine, Yokohama, Japan

[*] Corresponding authors: Kengo Azushima, M.D., Ph.D. and Hiromichi Wakui, M.D., Ph.D.

Department of Medical Science and Cardiorenal Medicine

Yokohama City University Graduate School of Medicine

3-9 Fukuura, Kanazawa-ku, Yokohama, Japan 236-0004.

Tel.: 81-45-787-2635; Fax: 81-45-701-3738

E-mail: azushima@yokohama-cu.ac.jp or hiro1234@yokohama-cu.ac.jp

# Full unedited gel for Figure 5D

GLP-1

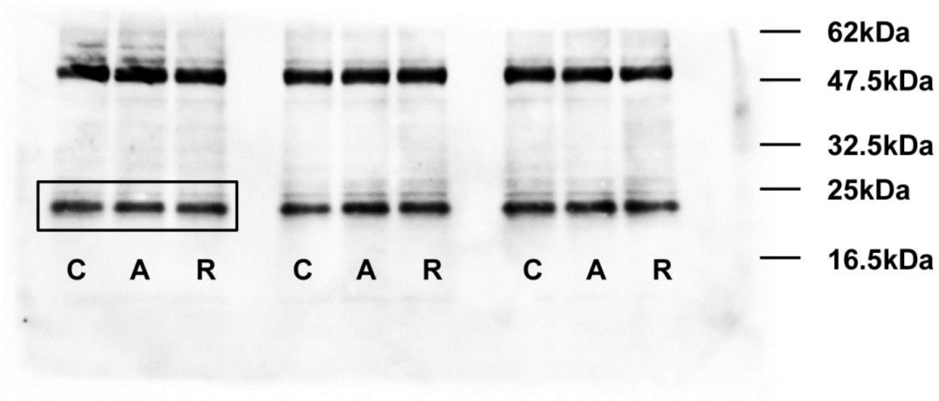


β-actin

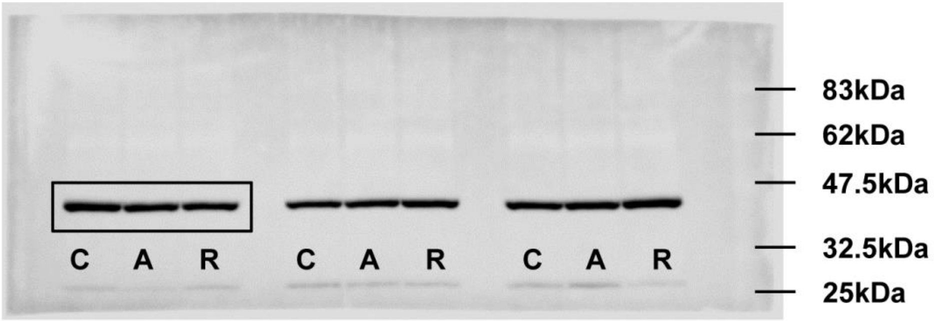


C, Control group; A, Ang II group; R, Ang II + RKT group.
